# The Latino Health Insurance Program: A Pilot Intervention for Enrolling Latino Families in Health Insurance Programs, East Boston, Massachusetts, 2006-2007

**Published:** 2009-09-15

**Authors:** Milagros Abreu, H. Patricia Hynes

**Affiliations:** Boston University, School of Public Health; Boston University, Boston, Massachusetts

## Abstract

**Background:**

Thirteen percent of Latinos in Massachusetts lack health insurance, the highest rate of any ethnic or racial group. Families without health insurance are more likely to be in poor or fair health, to lack a regular medical provider, and to not have visited a medical provider in the past year.

**Context:**

The Latino Health Insurance Program is designed as a response both to the high rate of uninsurance among Latinos in Boston and to the multiple obstacles that keep Latino parents from applying for insurance for their families.

**Methods:**

In 2006, we designed and implemented a culturally competent model of health insurance outreach, education, enrollment and maintenance, and referral for primary care and social services for Latino families.

**Consequences:**

Year 1 results of the Latino Health Insurance Program are promising. Six community members were hired and trained as case managers. A total of 230 children and adults were enrolled or re-enrolled in health insurance programs and received other needed services. Retention was near 100% after 1 year.

**Interpretation:**

The Latino Health Insurance Program may serve as a model health insurance access program that can be adapted by community-based organizations and also can be incorporated into public agency programs for Latinos and other immigrant and minority groups. The program continues to serve East Boston residents and was expanded in 2008.

## Background

More than 8.5 million children in the United States do not have health insurance, putting them at risk of untreated illnesses and lack of preventive care (1). Most uninsured children are poor and immigrant (2)*.*The proportion of Latino children who are uninsured is 21%, higher than for any other ethnic or racial group (1). In 1997, Congress created the State Children's Health Insurance Program (SCHIP) and allocated $39 billion to states to provide health insurance coverage for uninsured children. On December 29, 2007, President Bush signed legislation to extend SCHIP through March 2009, after having vetoed a bill that would have included an additional $35 billion over 5 years to cover more uninsured children. On February 4, 2009, President Obama signed a law expanding SCHIP to include 4 million uninsured children. Given the existence of this program, it is necessary to understand why so many Latino children are uninsured and what impediments they face to getting health insurance (3). Developing and testing models of insuring Latino children and their families in available health programs are essential and require knowledge of the obstacles that keep eligible families from enrolling in health insurance.

Massachusetts has many programs to reduce the number of residents without health insurance. Nonetheless, a larger percentage of Latinos (13%) than whites (5%) are uninsured (5%) (4). Latinos are the largest minority group in Massachusetts; they constitute more than 7% of the state population and almost 15% of the population of Boston (5). Demographic characteristics of many urban neighborhoods have changed substantially in the past 20 years. For example, in the East Boston neighborhood, the nonwhite population grew from 4% in 1980 to 50% in 2000, and nearly 40% of its residents are Latinos. Recent studies indicate that the rate of uninsured Latino children (37%) in East Boston is the highest in the city of Boston (3).

### Consequences of being uninsured

Not having health insurance has consequences for health, health care, and medical costs. Children without health insurance are more likely to be in poor or fair health, to lack a regular medical provider, and to not have visited a medical provider in the past year (6,7). They are more likely to use critical health care services and less likely to use primary and preventive health care services than are insured children (6,7). Uninsured parents often postpone seeking medical treatment for themselves and their children, which can have serious and costly consequences (8). A child treated early for uncomplicated appendicitis can be released from the hospital in 2 days, at a cost of approximately $8,000. A child admitted for a perforated appendix may have a hospital stay of 7 to 10 days, which costs an estimated $100,000 more (8).

Latinos without health insurance are more likely to use the emergency department than to visit a doctor's office (9). Unnecessary use of emergency services is responsible for high charges to the Massachusetts Health Safety Net, formerly known as the Uncompensated Care Pool, which pays for medically necessary services provided by hospitals and community health centers to low-income uninsured and underinsured residents. Costs charged to the Uncompensated Care Pool for a computed tomographic scan ranged from $359 to $4,401 in 2003 (10). In 2001, US hospitals provided $23.6 billion in uncompensated health care (11). The same year, providers submitted invoices for approximately $35 billion for which they received $30.6 billion from government sources, either through direct subsidies or reimbursement (11)*.*

Uninsured people die prematurely and experience poorer quality of services than do insured people (12). The Institute of Medicine estimates that approximately 18,000 deaths per year are attributable to lack of health insurance (12) because uninsured people are less likely to use preventive services, to have a regular source of care, and to benefit from early treatment and diagnosis. Latinos in the United States who are younger than 65 years and who have chronic diseases such as diabetes are less connected to the health care system than members of non-Latino groups and less likely to use it effectively or to feel confident in it to manage their medical conditions (9).

### Barriers to obtaining health insurance

Poor health literacy affects people who are older, immigrants, poor, or poorly educated (3). A recent study found that Latino parents in Boston had difficulty obtaining health insurance coverage for their children because of lack of information and misconceptions. Parents did not understand the eligibility criteria and application process for MassHealth (which administers Medicaid and other public health insurance programs for Massachusetts residents) or the Children's Medical Security Plan, which covers children who are not eligible for MassHealth, including those whose immigration status is undocumented (3). Parents also reported language barriers and the complexity of the application process as obstacles to applying (3)*.*

Another barrier to health obtaining insurance for immigrants is their concern about immigration status (13). Some have reported being apprehensive that using health benefits may diminish their chance of gaining citizenship or their relatives' likelihood of being allowed to immigrate to the United States (13). Fear of being reported to immigration authorities by a health service provider is another obstacle identified by undocumented Latino immigrants and one that would deter them from enrolling in health insurance (14).

## Context

We report the results of the 1-year pilot of the Latino Health Insurance Program (LHIP), a health insurance enrollment program that employs trusted local community leaders as case managers (14) and uses culturally specific methods of outreach and education in the Latino community of East Boston, Massachusetts. The LHIP is based on models that have been used successfully in other community health contexts to improve risk factors, health care use, and medical professional and lay communication (15,16). However, the program is unique in Massachusetts for its use of community health workers to enroll Latino immigrants in health insurance and connect enrolled families with needed services.

The program initially targeted East Boston because it is home to the largest Latino population in Boston. Our results are from the 1-year pilot of the LHIP pilot program, from April 2006 to May 2007.

## Methods

We describe the methods used to accomplish the following LHIP activities:

- Recruit and train community leaders as case managers in health insurance programs.
- Use culturally competent approaches to reach and recruit uninsured immigrants.
- Provide health insurance eligibility education and enrollment for recruited immigrant residents.
- Follow up with families to help them maintain health coverage and use primary care and other social and legal services.
- Engage local primary care providers and health agencies to create a network for primary care referrals.
- Document program results.

### Community leaders as case managers

The program director (M.A.) posted written notices to recruit case managers in 2 East Boston public housing developments. Referrals from community agencies were also used to identify and recruit residents with leadership qualities for the program. Recommendations from community members were given high consideration. The program director interviewed and selected case managers to ensure that the mix of case managers reflected the countries of origin of Latino residents. The program director organized training and ongoing inservice education for the case managers.

### Outreach with cultural specificity

The program team, consisting of case managers and the program director, visited public housing developments in East Boston, where they knocked on residents' doors and explained the LHIP. Families were invited to participate in educational sessions. The program team conducted similar outreach at community sites that are ideal for face-to-face information sharing, including bodegas, beauty salons, self-service laundries, restaurants, automobile repair shops, gas stations, and churches (14,17). All oral and written communication was available in Spanish and English.

### Education, enrollment, and follow-up

After outreach and recruitment began, educational sessions were held on 2 evenings per month for 5 consecutive months at trusted community locations (including 2 community rooms of the housing developments and local churches) and at times convenient for participants. Child care and a meal with ethnically appropriate food were provided. The program director led the sessions in Spanish with assistance from representatives of state advocacy agencies that work with immigrants and from state officials who discussed the new Massachusetts health law (18) and the 2 new health insurance plans offered by the state, Commonwealth Care and Commonwealth Choice (18).

Before the educational sessions, attendees were interviewed to determine whether they were eligible for health insurance. The educational session lasted 2 hours. Case managers helped eligible families who wished to enroll for health insurance to apply at the end of each session. Follow-up help was provided at a local church office if participants did not have the required documentation or the application was not completed at the session. The case managers scheduled one-on-one visits with participants, when necessary, to complete applications and collect required documentation; they then sent completed forms electronically to MassHealth for coverage determination. MassHealth determines who qualifies for state entitlement programs, including Medicaid, the Children's Medical Security Plan, Commonwealth Care, or the Health Safety Net (see Appendix for details on health insurance programs). Case managers completed online applications through the Commonwealth Connector Web site (www.mahealthconnector.org) for families and individuals with an income above 300% of the federal poverty level, who were not eligible for these 4 state entitlement programs.

### Network of referrals

The LHIP established direct communication with the East Boston Community Health Center and the Boston Public Health Commission to assure ease of referral and primary care provision for newly enrolled Latinos. The LHIP also linked residents with programs that address domestic violence, senior services, food stamps, fuel assistance, and legal services.

### Program documentation

The LHIP documented the following outcomes of its 1-year pilot:

- Number of children and families who were successfully enrolled or re-enrolled in public health insurance programs after parent educational sessions.
- Number of children and families who maintained their insurance during 7 months of follow-up and number of referrals to primary care providers.
- Number of referrals to other services.

## Consequences

### Community leaders as case managers

Thirteen East Boston Latino residents were recruited and interviewed by the program director. Of the 6 chosen as case managers, 2 had medical or clinical experience, 3 had community organizing experience, and 1 was a pastor. They came from the major Latin American subgroups in Massachusetts and East Boston and included 1 Puerto Rican, 3 Dominicans, 1 Salvadoran, and 1 Colombian. All case managers worked 8 hours per week.

The MassHealth educational unit trained case managers on MassHealth and the Children's Medical Security Plan, and the LHIP program director organized research-based training on the obstacles for Latino families to enroll in health care (3). Case managers then received 1 week of supervised on-the-job training by the program director. Inservice training consisted of ongoing supervision by the program director, forums on the new Massachusetts health reform law and its Commonwealth Care program, and updates on MassHealth.

### Outreach with cultural specificity

Knocking on doors at the East Boston housing developments and the face-to-face invitation to participate in the educational session at trusted community sites proved successful at attracting residents to the education and enrollment sessions. Local churches provided space for educational sessions and made referrals to the program, adding to the success of recruitment. An additional point of recruitment was the Salvadoran consulate; Salvadorans have been identified in previous studies as the most uninsured group in the East Boston neighborhood (17). The management offices of the East Boston housing development also referred families to the LHIP. Finally, word of mouth among enrolled residents brought other residents.

### Education, enrollment, and follow-up

During the year-long pilot program, 104 of 130 adults and all 100 children were determined to be eligible and were enrolled in state-subsidized and nonsubsidized health insurance programs, the largest subgroups being Dominican and Salvadoran by country of origin and citizens and undocumented immigrants by civic status (Table 1). All children were enrolled in either MassHealth or the Children's Medical Security Plan. More than 70% of the adults signed up for health insurance programs after educational sessions. The remainder signed up during office visits to the LHIP after being referred by other participants. Twenty percent of the adults were ineligible for any type of insurance coverage, and they were assisted in receiving services through the Uncompensated Care Pool offered at their local health center or hospital (Table 2).

### Network of referrals

A total of 123 households were assisted with obtaining primary care providers and social services. Newly enrolled East Boston residents who did not have a provider were referred to the East Boston Neighborhood Health Center to choose a health care provider for their children and themselves and to be seen for primary care, if necessary. The case managers referred newly insured residents from outside the East Boston neighborhood to the Boston Public Health Commission for primary care and for additional programs when needed, such as domestic violence shelter or senior services. Case managers also electronically enrolled eligible participants into the state food stamp program, now known as the Supplemental Nutrition Assistance Program. For fuel assistance, participants were referred to a local agency that helps eligible families pay their electric and gas bills during winter; for legal representation in medical disputes, especially given the uncertainty about the new health law in Massachusetts, they were referred to Health Law Advocates, pro bono attorneys. Case managers helped residents to apply for Social Security assistance for their eligible disabled children and to enroll in citizenship classes if they wished to change their immigration status (Table 3).

### Program documentation

The LHIP documented the numbers and characteristics of enrollees, the types of health insurance received, and referrals for health and social services (Tables 1-3). The program tracked families as they moved and maintained follow-up with nearly all participants over the course of the year to assure that they stayed enrolled in health insurance plans and that they received needed services.

## Interpretation

The efforts of Massachusetts and Boston to insure Latino immigrants must overcome fear, inadequate information, low wages, lack of employers that offer health insurance to Latino residents, and frequent change of address. Many Latinos are fearful of government because of their immigration status (13). Others lack adequate information about eligibility requirements for public insurance programs, given the complexity of the requirements and the reluctance of Latinos to seek out government authorities for information (3). Additional impediments to obtaining health insurance reported by Latino clients who enrolled through the LHIP are that they work for low wages for employers that do not offer medical coverage and that they cannot afford to pay for it themselves. Even if they worked for employers that offer medical insurance, some LHIP participants reported that they could not afford their portion of insurance premiums. Some participants reported being afraid to accrue high medical bills and avoided seeking medical care. Others reported that they thought working full-time made them ineligible to apply for publicly supported medical insurance for themselves or their children.

Inability to track enrollees because of change of address has been a major issue for Boston's health insurance enrollment program, known as the Mayor's Health Line. Applications are often closed without final approval because of the difficulty in collecting the necessary documentation for MassHealth to make the final determination of eligibility for health coverage (19). The primary reason that MassHealth stops medical coverage is the inability of the insured party to verify current Massachusetts residency (Appendix). Some families in the LHIP reported having moved 5 times in less than 1 year.

The case manager's rapport with families, specifically, using well-trained and trusted community members, is a key component of the LHIP's success. Other elements in the LHIP that appear to have overcome many of these obstacles to create a successful insurance enrollment program for Latinos include

- Conducting educational outreach in relevant community places.
- Holding sessions at convenient times for participants.
- Providing child care and a meal at sessions.
- Developing a network of referrals based on the needs of participants.
- Sustaining contact with the newly enrolled family.

These are methods used successfully by community health workers in other health contexts (15,16). The LHIP has employed them with good results for health insurance outreach and enrollment, primary care referral, and sustained follow-up.

### Conclusion and recommendations

We cannot generalize from this pilot intervention program in the Latino community of East Boston to other underinsured populations of racial and ethnic minorities, particularly regarding the methods and messages of outreach and recruitment. All populations with high rates of underinsurance should be reached by using models, methods, and messages that are culturally relevant. We suggest that the optimal programs to fulfill government responsibility for assuring access to state-sponsored programs are ones that arise from partnerships between a municipal health agency and community-based advocacy groups.

During the pilot program, the LHIP partnered with the Boston Public Health Commission, the Massachusetts Immigration and Refugee Advocate Coalition, Health Law Advocates, and the East Boston Neighborhood Health Center to ensure that newly insured Latinos were linked with local health providers and social and legal services and advocates. These partnerships also provide the health agencies and institutions with a culturally and linguistically competent community partner in the mutual goal of improving primary health care and social services to all community members. After the pilot program, the LHIP model was adapted by the Boston Public Health Commission for use in Safe Shops, an occupational and environmental health education and outreach program to Latino-owned automobile repair shops. The LHIP also trained Massachusetts members of the Brazilian American Association to use its model in the Brazilian community. The program continues to serve East Boston residents and was expanded in 2008 to Framingham, in suburban Boston.

Local health agencies, health care providers, and foundations with a family health mission can partner with community-based programs such as the LHIP that are more strategic, proficient, and nimble in outreach, education, and recruitment. With culturally competent partners, health agencies and providers can focus on providing effective primary care and prevention programs for newly insured residents.

## Figures and Tables

**Table 1:** Sociodemographic Characteristics, Immigrant Status, and Insurance Status of Participants in the Latino Health Insurance Program Pilot, East Boston, Massachusetts, 2006-2007

**Characteristics**	**Adults (n = 130)**	**Children (n = 100)**
Mean age, y	33	6
Latino subgroup, %		
Dominican	37	18
Salvadorian	37	10
Colombian	16	10
Puerto Rican	8	0
United States-born	0	60
Other	2	2
Citizenship status, %		
Citizens	32	76
Green card holders	19	13
Undocumented immigrants	38	11
Work permit holders	11	0
Single-parent household, %	65	NA
Mean annual household income, $	11,110	NA
Limited English proficiency^a^, %	90	NA
Community of residence, %		
East Boston	56	75
Chelsea	11	8
Revere	10	6
Other	23	11

Abbreviation: NA, not applicable.

a English proficiency was assessed by case workers.

**Table 2:** Type of Health Insurance Coverage for Participants in the Latino Health Insurance Program Pilot, East Boston, Massachusetts, 2006-2007

**Type of Coverage^a^**	**Adults, % (n = 130)**	**Children, % (n = 100)**
MassHealth (Standard or Family Assistance)	40	86
Children's Medical Security Plan	NA	14
Commonwealth Care and Commonwealth Choice	40	0
Uncompensated Care Pool (Health Safety Net)	20	0

Abbreviation: NA, not applicable.

a For a detailed explanation of coverage types, see the Appendix.

**Table 3:** Referrals for Primary Care and Services for Participants in the Latino Health Insurance Program Pilot, East Boston, Massachusetts, 2006-2007

**Types of Referrals**	**No. of Newly Enrolled Participants**	**Key Outcomes and Barriers**	**Comments**
Primary care providers	36 adults, 12 children	Some residents who had not had a primary care provider since coming to the United States were assigned a doctor in 1 day after enrolling in LHIP.	Parents and their children had lived in the United States without seeing a doctor for 6 months to 25 years. One child was diagnosed with lead poisoning after being enrolled in an insurance program.
Food stamps	29 adults, 37 children	Many Latino children and adults were eligible for food stamps after parents applied for the program through the LHIP.	Some adults who had been living in Massachusetts for 25 years did not know about the food stamp program.
Emergency food distribution center	10	All of those referred (who were not eligible for food stamps) received food in convenient community locations.	Three disabled adults got food after the LHIP arranged help and transportation.
Health Law Advocates (HLA)	20	People who needed help with medical debts to health care providers and payers before they could be insured were referred to HLA.	One mother had an outstanding bill of $2,000 that was reduced to $100 after she received legal representation. A parent with a disabled child received legal representation when she was sued by her child's dentist for inability to pay.
Fuel assistance	10	Participants were referred to East Boston APAC for assistance with gas and electrical bills.	Some families received more than 1 service, including fuel assistance.
Domestic violence shelter	2	Women who reported being verbally or physically abused by partners were referred to domestic violence shelters.	Cases are under legal representation and custody.
Social Security assistance for disabled children	2	Parents of severely disabled children with cerebral palsy had never collected assistance because of earlier denials.	Parents are now collecting Social Security assistance for their disabled children who qualify.

Abbreviations: LHIP, Latino Health Insurance Program; APAC, Area Planning Action Council (now the Action for Boston Community Development).

## Appendix

### Appendix

#### MassHealth

MassHealth is the name used in Massachusetts for Medicaid and the State Children's Health Insurance Plan (SCHIP), combined in 1 program. It is funded by the state and federal governments and covers eligible low- and middle-income residents of the state.

MassHealth includes several coverage types with different rules for each type:

- MassHealth Standard: comprehensive health insurance, including long-term care, for low-income Massachusetts residents including parents with children younger than 19 years, pregnant women, children up to age 19 years, the elderly, and disabled people.
- CommonHealth: coverage similar to MassHealth Standard for eligible disabled adults and disabled children younger than 19 years who cannot get MassHealth Standard because their family incomes are too high.
- MassHealth Family Assistance: health insurance with most of the services of MassHealth Standard for children younger than 19 years and people with HIV who are not eligible for MassHealth Standard or CommonHealth; also helps pay premiums for eligible employed adults.
- MassHealth Basic: health care benefits or premium assistance for recipients of Emergency Aid to Elders, Disabled and Children, and low-income clients of the state Department of Mental Health who are long-term unemployed.
- MassHealth Limited: emergency medical coverage for noncitizens whose immigration status makes them ineligible for other MassHealth programs.
- Medicare Buy-In programs: programs that pay all or part of Medicare health insurance expenses for eligible low-income Medicare recipients.
- MassHealth Prenatal: short-term outpatient prenatal care (does not include labor and delivery) for low-income pregnant women.
- MassHealth Essential: coverage is similar to MassHealth Basic, but more limited.

Source: http://www.massresources.org/pages.cfm?contentID=35&pageID=13&Subpages=yesMassHealth.

#### Children's Medical Security Plan (CMSP)

The CMSP provides primary and preventive medical and dental coverage to uninsured children who do not qualify for MassHealth (except MassHealth Limited, which covers emergency services). The program is for children younger than 19 years who are Massachusetts residents at any income level. There may be a waiting list to receive CMSP coverage. Children covered by CMSP with family incomes up to 400% of the federal poverty level are eligible for the Health Safety Net (formerly known as the Uncompensated Care Pool) at Massachusetts acute hospitals for inpatient services not covered by CMSP. Deductibles and premiums may apply, based on family size and income.

Source: http://www.mass.gov/?pageID=eohhs2terminal&L=4&L0=Home&L1=Consumer&L2=Insurance+(including+MassHealth)&L3=Additional+Insurance+and+Assistance+Programs&sid=Eeohhs2&b=terminalcontent&f=masshealth_consumer_additional_ier_cmsp&csid=Eeohhs2

#### Commonwealth Care

Commonwealth Care is for low- and moderate-income Massachusetts residents aged 19 years or older who do not have health insurance. Members get free or low-cost health services through managed care health plans offered by private health insurance companies. Members can choose among the plans, which have different costs. Commonwealth Care is for adults who are not eligible for MassHealth programs. Undocumented noncitizens cannot get Commonwealth Care.

Source: http://www.massresources.org/pages.cfm?contentID=81&pageID=13&Subpages=yes#whatis

#### Commonwealth Choice

Commonwealth Choice is for uninsured Massachusetts residents who are 19 years or older. The program offers unsubsidized health insurance to people who are not eligible for MassHealth or Commonwealth Care. Commonwealth Choice health plans are for adults who do not qualify for Commonwealth Care because their family income is higher than 300% of the federal poverty level. There are no income or asset limits for the program.

Source: http://www.massresources.org/pages.cfm?contentID=85&pageID=13&Subpages=yes#whatis

#### Health Safety Net (Free Care)

The Health Safety Net replaced the Uncompensated Care Pool (also called Free Care) on October 1, 2007, as a program for Massachusetts residents who are not eligible for health insurance or who cannot afford it. To be covered by the Health Safety Net, Massachusetts residents must be uninsured or underinsured and have no access to affordable health coverage. People of any income with large medical bills that they cannot pay are also eligible. Citizenship or immigration status does not affect eligibility. The Health Safety Net pays all or some of the medically necessary services at Massachusetts community health centers and hospitals, depending on age and income. To be covered, services must be on the MassHealth Standard list of covered services.

Source: http://www.massresources.org/pages.cfm?contentID=50&pageID=13&Subpages=yes
